# FLRT2 functions as Tumor Suppressor gene inactivated by promoter methylation in Colorectal Cancer

**DOI:** 10.7150/jca.47558

**Published:** 2020-10-23

**Authors:** Xiaohong Guo, Chao Song, Lei Fang, Min Li, Longtao Yue, Qing Sun

**Affiliations:** 1 Department of Pathology, The First Affiliated Hospital of Shandong First Medical University & Shandong Provincial Qianfoshan Hospital, Jinan, Shandong, China.; 2 Department of Pathology, Zibo Central Hospital, Zibo, Shandong, China.

**Keywords:** FLRT2, DNA methylation, colorectal cancer, tumor suppressor

## Abstract

Colorectal cancer (CRC) is a leading cause of cancer death worldwide. Epigenetic alterations, especially DNA methylation, contribute to the initiation and progression of CRC. To identify novel methylated tumor suppressors in CRC, MethylRAD-Seq screening was performed. As the result, FLRT2 was found to be preferentially methylated. In the present study, we aimed to elucidate the epigenetic regulations and biological functions of FLRT2 in CRC. Significant FLRT2 hypermethylation was initially confirmed in CRC samples and cell lines. Meanwhile, downregulated expression of FLRT2 was observed in CRC, which is probably attributed to promoter methylation of FLRT2. Consistently, the expression of FLRT2 was restored after treatment with DNA demethylating agent 5-AZA. FLRT2 overexpression resulted in impaired cell viability and colony formation. Additionally, FLRT2 overexpression led to a reduction in cell migration and cell invasion. Furthermore, we also observed that FLRT2 induced cell cycle arrest. Mechanistically, these effects were associated with the downregulation of phosphor-AKT, phosphor-ERK, CDK2, Cyclin A, and MMP2, and upregulation of P21. Taken together, these results define a tumor-suppressor role of FLRT2 with epigenetic silencing in the pathogenesis of CRC. Moreover, FLRT2 promoter methylation may be a useful epigenetic biomarker for the prevention and treatment of CRC.

## Introduction

CRC is the third most common cancer worldwide in both sexes with high incidence and mortality. For 2018, International Agency for Research on Cancer (IARC) estimated an incidence of ~1.8 million new cases of CRC (~10.2% of all cancers) and ~881,000 CRC-related deaths worldwide (~9.2% of all cancer-related deaths) 1. Although the expanded screening use of colonoscopy and the introduction of innovative treatments have improved clinical outcomes, CRC remains one of the leading causes of cancer-related death. The pathogenic mechanisms underlying CRC development appears to be complex with the concurrent accumulation of genetic and epigenetic alterations, resulting in the transformation of normal colonic epithelium into colorectal adenomas 2, 3.

Epigenetic changes, notably DNA methylation, play central roles in the pathogenesis of various cancers, including CRC 2. Changes in DNA methylation, with accompanied epigenetic gene silencing, appear to be the earliest somatic genomic alterations recognized in CRC and continue throughout disease progression 2, 4, 5. In addition, DNA methylation has been used as biomarkers that could eventually become very useful in predicting clinical outcomes or in prognostication of CRC 3, 6-9. Thus, the discovery of novel methylated tumor suppressors especially that exert vital roles in CRC development will not only help understand the pathogenesis of CRC, but also find a way to improve the early diagnosis for CRC.

In order to identify dysregulated genes by abnormal methylation in CRC, the MethylRAD-Seq screening was performed. Through this survey, a set of methylated genes were identified, one of which is FLRT2 (Fibronectin leucine rich transmembrane protein 2).

FLRT2 is a member of FLRT family, which are characterized by a conserved 10 LRR (leucine-rich repeats) domains and a FNIII (type III fibronectin) domain, followed by the transmembrane segment with a short intracellular tail 10. Most studies about FLRT2 so far were focused on its physiological roles on the vascular, neural and embryonic development 11-16. FLRT2 was reported to contribute to the altered cellular functions or phenotypes during development via interacting with several proteins such as UNC5, fibronectin, and FGFR 17-20. Limited studies have explored the role of FLRT2 on tumorigenesis process. FLRT2 was found to be potentially associated with the process of ovarian and uterine cancers due to downregulated expression 21-23. Its biological function was verified only in prostate cancer and breast cancer 24, 25, but its role in the tumorigenesis of CRC remained unclear.

In the present study, FLRT2 promoter was found hypermethylated in CRC. Moreover, the aberrant promoter methylation of FLRT2 was correlated well with its decreased expression which could be successfully restored by treatment with DNA demethylating agent 5-AZA. Further investigations showed that FLRT2 negatively regulate cell proliferation and migration through AKT and ERK signaling pathway. In conclusion, our findings define that FLRT2 was a putative tumor suppressor in CRC and a potential biomarker for CRC diagnosis and therapeutic applications.

## Materials and methods

### Human tissue samples and cell lines

A total of 51 cases of paraffin-embedded CRC specimens were obtained from the archives of the Department of Pathology of the First Affiliated Hospital, Shandong First Medical University between 2016 and 2019. 22 cases of paired fresh CRC and adjacent noncancerous colon tissues were collected from patients at the time of surgical resection for the determination of FLRT2 mRNA and protein expression. The study was approved by the Medical Ethics Committee of the First Hospital Affiliated to Shandong First Medical University (Shandong Provincial Qianfoshan Hospital). Patients with colorectal cancer were confirmed according to the World Health Organization's morphological criteria. The study population was selected according to the following criteria.

The inclusion criteria: I) pathological diagnosed with CRC; II) CRC samples were approved for experimental analysis; III) detailed clinicopathological data was available. The exclusion criteria: I) incomplete patient information; II) with a history of other tumor diseases, or with other genetic diseases; III) preoperative chemotherapy, radiotherapy or immunotherapy.

Normal colorectal cell (FHC) and seven CRC cell lines (LOVO, CaCO2, HT29, HCT116, SW480, SW620, and SW1463) used in this study were obtained from ATCC. FHC, LOVO, CaCO2, HT29, and HCT116 were cultured in DMEM supplemented with 10% fetal bovine serum (FBS) and 1% Penicillin/Streptomycin. SW480, SW620, and SW1463 were cultured in RPMI-1640 medium supplemented with 10% FBS and 1% Penicillin/Streptomycin. All cell lines were cultured at 37 °C with 5% CO2.

### Methylation-specific PCR (MSP)

Genomic DNA was purified from FFPE and fresh-frozen human tissues using TIANamp Genomic DNA Kit (Tiangen, China). 1.5 µg DNA was used for conversion of methylation based by using EpiTect Bisulfite Kit (Qiagen, Germany). MSP primers were designed to amplify the methylated and unmethylated alleles for FLRT2, and the amplified MSP products were subjected to gel electrophoresis. The primer and their sequences were as follows: FLRT2 methylation primer (5'-TCGCGGCGTTTATATTAGTTC-3', 5'-AATTTAAAAAATCCCGAAACTCG-3'), FLRT2 unmethylation primer (5'-GGGTTGTGGTGTTTATATTAGTTTG-3', 5'-AATTTAAAAAATCCCAAAACTCAAC-3').

### Quantitative PCR analysis

To measure gene expression in CRC tissues and cell lines, total RNA was isolated with TRIzol reagent (Invitrogen, USA), and reverse transcription was performed using ReverTra Ace (Toyobo, Osaka, Japan) according to the manufacturer's instructions. Real-time qPCR was performed on a 7500 Real-Time PCR System (Applied Biosystems, USA) using UltraSYBR Green Master Kit (CWBIO, China). The mRNA levels were calculated using the ΔΔ*Ct* method. All qPCR primers and their sequences were as follows: FLRT2 (5'-ACCCTTGGTTTTGTGACTGC-3', 5'-AGGACCTTGGCACATGAAAC-3') and β-actin (5'-CATGTACGTTGCTATCCAGGC-3', 5'-CTCCTTAATGTCACGCACGAT-3').

### Western blot analysis

Total proteins were isolated from fresh tissues and cells using RIPA buffer containing protease inhibitor PMSF. Protein concentration was quantified by a bicinchoninic acid (BCA) protein quantification kit (Beyotime, China). Equal amounts of protein were separated by SDS-PAGE and transferred onto nitrocellulose membranes (Pall, Port Washington, NY) for immunoblotting with primary antibodies as indicated. The antibodies for immunoblot analysis were shown as follows: phospho-PDK1, AKT, phospho-AKT, ERK, phospho-ERK and P21 antibodies were from Cell Signaling Technology, Beverly, MA. Cyclin A, CDK2 and MMP2 antibodies were from Abcam, Cambridge, MA. And β-actin antibody was from Beyotime Biotech, China. β-actin were used as a loading control. The blots were visualized using enhanced chemiluminescence (Cell Biosciences, USA).

### 5-Aza-2'-deoxycytidine (5-AZA) treatment

CRC cell lines were seeded into 6-well plates at a density of 2×10^5^ cells/well, then treated with culture medium conditioned with 0, 2.5, 5 and 10 μM 5-AZA (Sigma, USA) for 3 days. The fresh medium containing 5-AZA was replaced daily. Total RNA was isolated for qPCR analysis. The control samples were treated with DMSO only.

### Lentiviral infection

HCT116 and SW480 cells were infected with either vector-only control or FLRT2-overexpressing lentivirus (GeneChem, China). After 12 h, the culture medium was replaced with fresh virus-free medium and incubated for another 72 h. Then the cells were harvested for further experiments.

### Cell proliferation and colony formation assays

After infection with the lentiviruses, the cells were used for cell growth and colony forming assay. Cell proliferation was measured in accordance with the manufacturer's instructions at 0, 24, 48, and 72h (MTT kit, Beyotime, Nanjing, China). For colony formation assay, 1000 cells were plated into 3.5 cm-diameter petri-dishs. After 14 days, cells were fixed with 4% paraformaldehyde solution for 30 min, stained with 0.5% crystal violet (Beyotime, Nanjing, China) for 15 min, and colonies were counted visually under miscroscope.

### Transwell migration and invasion assays

For cell migration analysis, 2×10^5^ cells were directly seeded onto 8 μM transwell filters (Corning, Corning, NY) and induced to migrate toward medium containing 10% FBS for 24 h. For cell invasion assays, the filter inserts were first coated with Matrigel prior to seeding the cells, and the rest of experimental protocol was similar to migration assays. Generally, 2×10^5^ serum-starved cells were plated into the upper chamber. Non-invading cells were removed with a swab. The remaining cells were fixed in 4% paraformaldehyde, stained with crystal violet, and quantified.

### Cell cycle assay

Following 72 h incubation, HCT116 and SW480 cells infected with either vector-only control or FLRT2 -overexpressing lentivirus were harvested. Cells were washed twice with cold PBS, fixed with ice-cold 70% ethanol overnight at 4 °C. Then, the fixed cells were stained with PI (Beyotime, china) containing RNase A at 37 °C for 30 min in the dark and sorted by Beckman coulter cell Lab Quanta SC. The cell phase distribution (G0/1, S, G2/M) was analyzed by Kaluza software.

### Statistical analysis

Χ^2^ test was used to evaluate relationships between FLRT2 gene methylation and clinicopathological parameters of patients with CRC. All results were expressed as the mean ±SEM. The* P* value was calculated using GraphPad Prism 5 statistical program and determined by two-tailed Student's *t*-test. Data was plotted by using GraphPad Prism 5 and SPSS software. A *p* value < 0.05 was considered statistically significant.

## Results

### FLRT2 is hypermethylated in human primary CRC and cell lines

**Figure 1:**
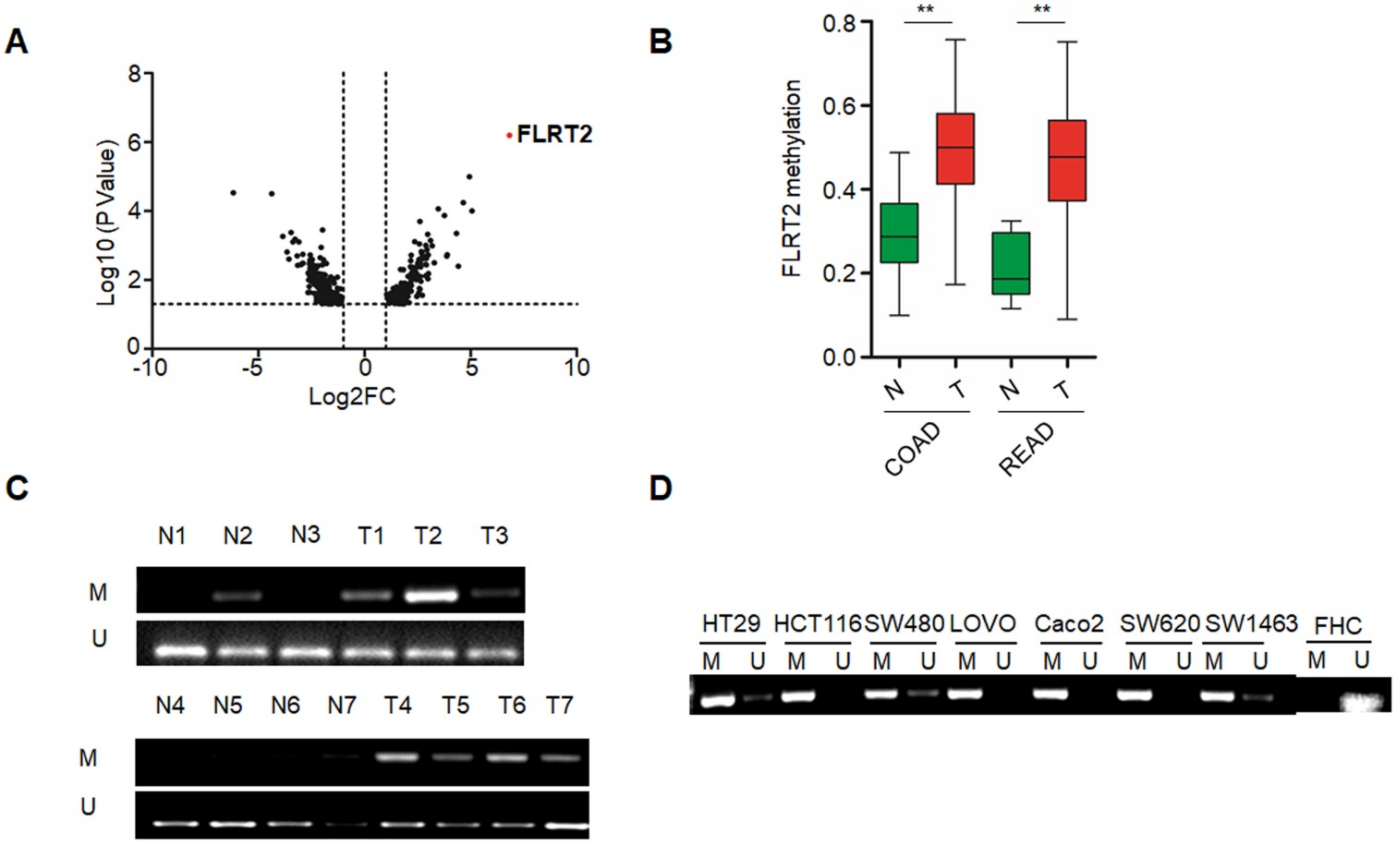
** Abnormal FLRT2 gene methylation in CRC tissues and cell lines.** (**A**) Volcano plot for gene methylation analysis data sets. Each dot represents an individual gene. The x-axis represents the difference of methylation value between the comparisons. The positive value indicates more methylation and a negative value indicates less methylation. The y-axis shows the p-value. The red dot was the top-ranked methylated gene FLRT2. (**B**) FLRT2 methylation status in colon adenocarcinoma (COAD, n = 274) and rectum adenocarcinoma (READ, n = 95) from MethHC database. (**C**) Assessment of FLRT2 methylation status in representative CRC samples (T) and matched adjacent normal tissues (N) by MSP assay. (**D**) The methylation state of FLRT2 was detected by MSP analysis in normal colorectal cell FHC and CRC cell lines. Error bars, SEM. M, methylated. U, unmethylated. **P < 0.01.

To identify the novel abnormal methylated genes in CRC, we initially performed a comparative genome-wide analysis for methylated loci in CRC samples paired with non-cancerous samples by exploiting MethylRAD-Seq method. Among potentially cancer-associated methylated genes identified, FLRT2 was one of top-ranked (Figure 1A). Further, genomic analysis of publicly available MethHC databases revealed that the methylation levels of FLRT2 were remarkably increased in colon adenocarcinoma (COAD) and rectum adenocarcinoma (READ) tissues (Figure 1B). These results correlated well with our sequencing results. To validate the methylation pattern, the methylation state of FLRT2 was assessed by MSP in CRC tissues and cell lines. Significant higher methylation of FLRT2 was observed in CRC tissues (32/51, 62.7%) than in normal colorectal mucosa (13/51, 25.5%) (Figure 1C). Consistently, abnormal methylated FLRT2 was also found in a panel of CRC cell lines (N=7), while the methylation of FLRT2 was not observed in normal colorectal cell FHC (Figure 1D). Together, these data indicate that FLRT2 is markedly methylated in CRC.

### FLRT2 expression is significantly correlated with promoter region hypermethylation in CRC

**Figure 2:**
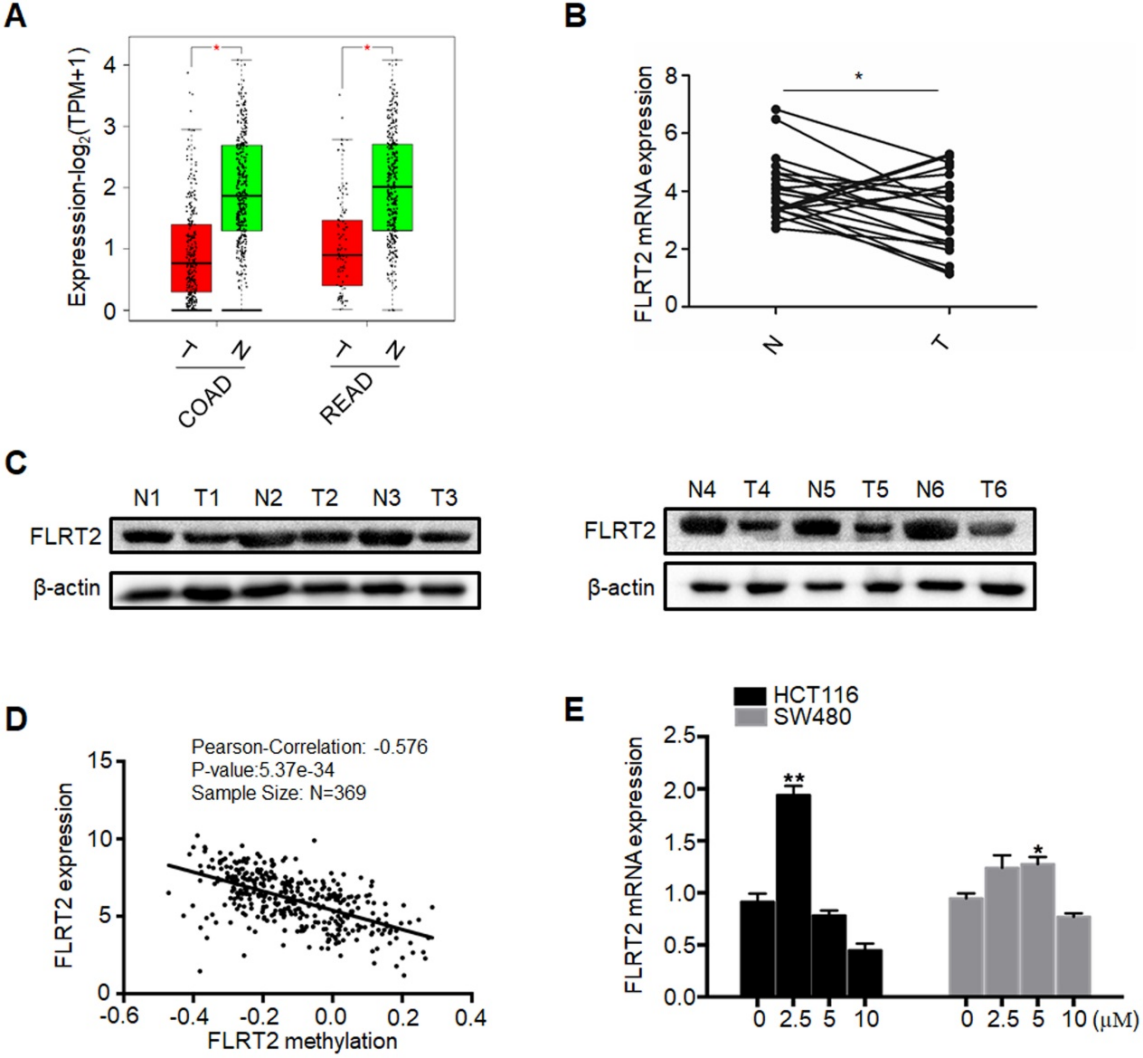
** Promoter methylation correlates with reduced expression of FLRT2.** (**A**) Relative expression of FLRT2 in COAD (num(T)=275; num(N)=349) and READ (num(T)=92; num(N)=318) from GEPIA database. The expression levels of FLRT2 in representative CRC cases and matched adjacent normal controls were determined by qPCR (**B**) and western-blot analysis (**C**). (**D**) Association of FLRT2 methylation and expression in CRC tissues. Scatter plots in 369 CRC samples from TCGA database were shown. (**E**) HCT116 and SW480 were treated with DNA methyltransferase inhibitor 5-AZA (0, 2.5, 5, 10 µM) for 3 days, and the expression of FLRT2 was measured by qPCR. Error bars, SEM. *P < 0.05, **P < 0.01.

To investigate whether DNA methylation regulated the expression of FLRT2, the expression analyses from GEPIA (Gene Expression Profiling Interactive Analysis) database were initially performed, revealing that FLRT2 expression was evidently reduced in COAD and READ tissues (Figure 2A). Subsequently, the expression level of FLRT2 was further examined in 22 CRC tumor samples and matched normal epithelial mucosa. Consistent with the aforementioned gene expression profiling data, a substantial decrease in FLRT2 expression was observed in 16 cases of CRC tissues compared with the adjacent normal tissue (Figure 2B). As shown in Table 1, among the 16 cases of CRC tumor samples with down-regulated FLRT2 expression, 13 cases exhibited FLRT2 methylation. However, of 6 cases of CRC tumor samples with up-regulated FLRT2 expression, only one cases displayed methylation. Similarly, the attenuated FLRT2 levels in CRC samples were further confirmed, as measured using western-blot assay (Figure 2C).

To further confirm the association between methylation status and the expression levels of FLRT2, the Cancer Genome Altas (TCGA) colon database was interrogated by LinkedOmics, which observed a close association in 369 cases of CRC tissue (R=-0.576, *P* < 0.001) (Figure 2D). Next, the CRC cell lines HCT116 and SW480, both with methylated FLRT2, were exposed with 5-AZA, which is a kind of DNA demethylating agent. As expected, the expression of FLRT2 was restored after 5-AZA treatment (Figure 2E). Overall, these results suggest that the expression of FLRT2 was regulated by promoter region methylation.

### Association between clinicopathological characteristics and FLRT2 methylation status

The DNA methylation patterns have frequently been regarded as promising clinical markers for the prevention and management of patients with CRC. Thus, the relevance between clinicopathological characteristics and FLRT2 methylation was investigated in 51 tissue specimens from CRC patients. As shown in Table 2, there was no correlation between FLRT2 hypermethylation status and clinicopathological characteristics in term of age, gender, tumor size or tumor location in 51 tissue specimens. However, FLRT2 methylation state was highly related to tumor stage and metastasis. This observation strongly suggests that FLRT2 methylation could be a useful indicator of clinical outcome.

### FLRT2 suppresses CRC cell proliferation

**Figure 3:**
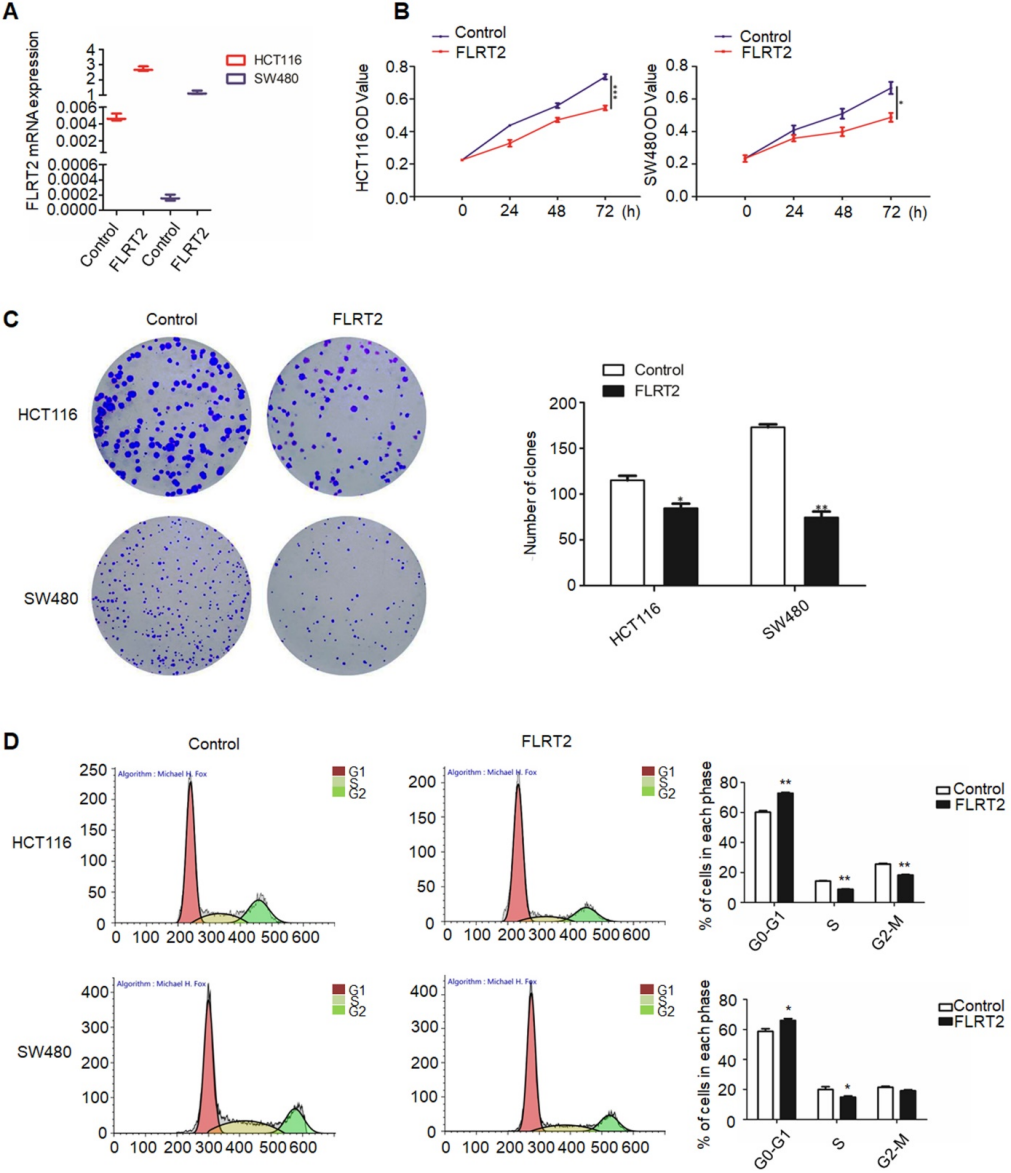
** FLRT2 inhibits CRC cell growth.** After infected with FLRT2 overexpression lentivirus, ectopic expression of FLRT2 in HCT116 and SW480 was validated by qPCR assay (**A**). (**B**) The overexpression of FLRT2 suppressed cell viability, as determined by MTT assays. (**C**) Left, the ability of colony formation in FLRT2-overexpressed cells decreased compared with control. Right, quantitative analysis of colony formation. (**D**) Left: cell cycle distribution of vector- and FLRT2-transfected HCT116 and SW480 cells was detected by flow cytometry analysis. Right: quantitative analysis of percent of cells in cell cycle phase. Error bars, SEM. *P < 0.05, **P < 0.01, ***P < 0.001.

Given on the above results, it is presumed that FLRT2 exerts functional roles in CRC progression. Therefore, FLRT2 was overexpressed using lentivirus-mediated system in HCT116 and SW480 cells. Meanwhile, the lentiviral overexpression of FLRT2 was ascertained by quantitative PCR analysis (Figure 3A). By MTT assays, ectopic expression of FLRT2 induced a decrease in cell proliferation (Figure 3B). And the results were further supported by colony formation assays. Cells overexpressing FLRT2 led to fewer colonies compared with the control (Figure 3C). Given the antiproliferative effect of FLRT2, its potential influence on cell cycle was detected by flow cytometry. Cell cycle analysis showed G0/1 phase was increased, whereas S and G2/M was decreased in CRC cells transfected with FLRT2 (Figure 3D). Collectively, these findings indicate that the suppressive role of FLRT2 in CRC proliferation was likely mediated by cell cycle arrest.

### FLRT2 inhibits CRC cell migration and invasion

**Figure 4:**
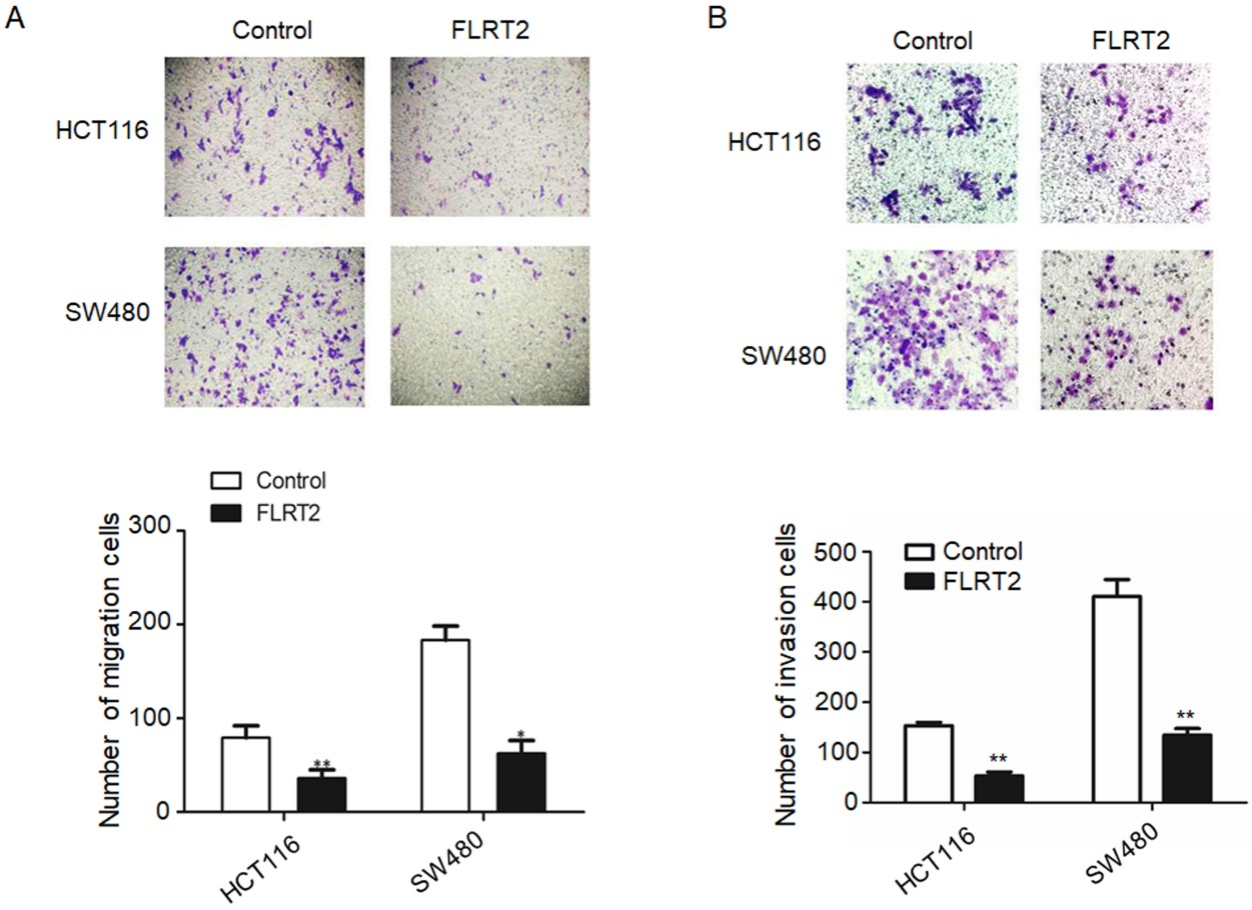
** FLRT2 suppresses cell migration and invasion.** After infected with FLRT2 overexpression lentivirus, the ability of cell migration (**A**) and invasion (**B**) were assessed by transwell assays in HCT116 and SW480 cells and quantitative analysis of the number of migrating and invasion cells. Error bars, SEM. *P < 0.05, **P < 0.01.

To identify the potential effect of FLRT2 on cell migration and invasion, transwell assays were performed. Overexpression of FLRT2 in HCT116 and SW480 cells was found to cause a significant reduction in cell motility (Figure 4A and 4B).

### FLRT2 regulates AKT and MAPK signaling pathway

**Figure 5:**
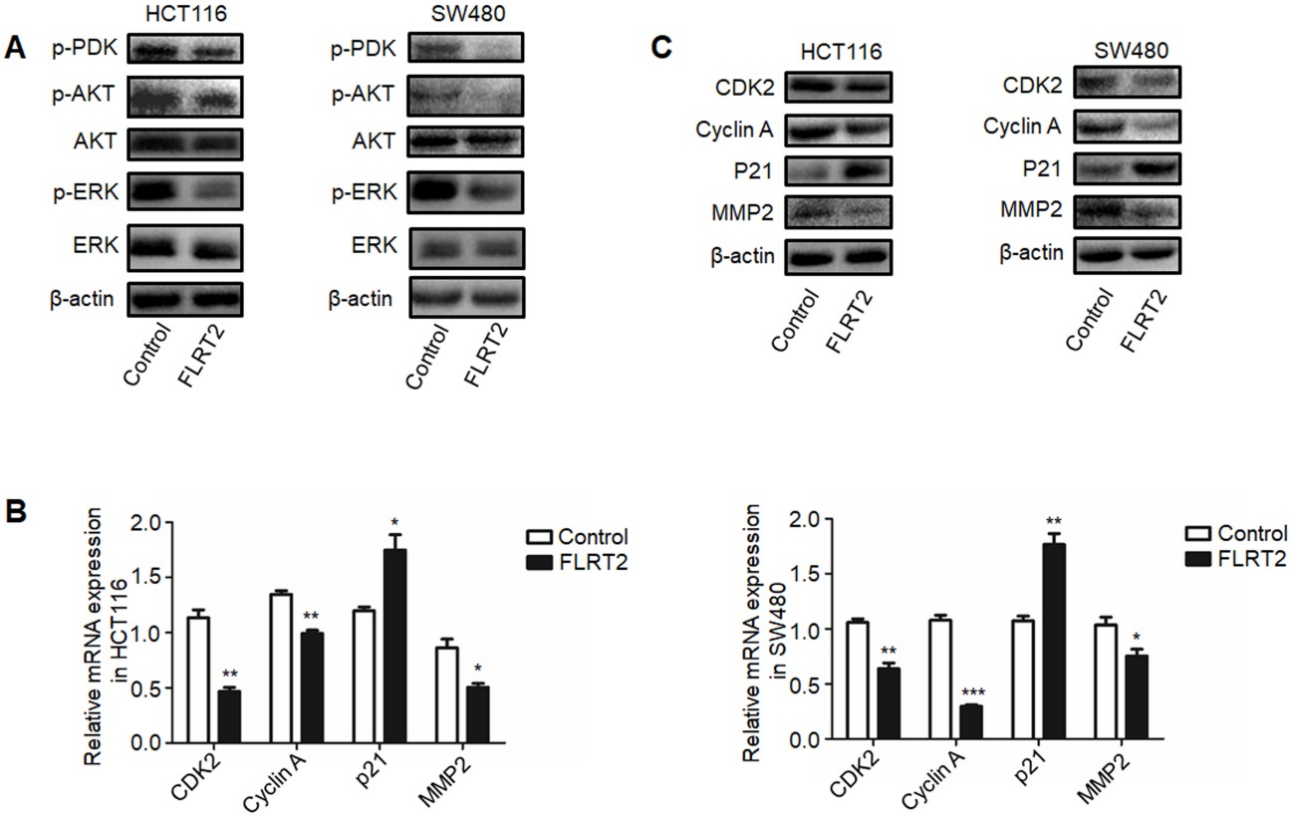
** FLRT2 negatively regulates PI3K/AKT and ERK signaling pathways.** The CRC cells (HCT116 and SW480) were infected with FLRT2 overexpression lentivirus. (**A**) Protein expression levels of phospho-PDK, phospho-AKT, AKT, phospho-ERK, and ERK were determined by western blot analysis. (**B and C**) Cell cycle markers and MMP2 were evaluated by qPCR and western blot assay. Error bars, SEM. *P < 0.05, **P < 0.01, ***P < 0.001.

It has been demonstrated that FLRT2 can interact with FGF receptors, thereby modulating PI3K/AKT and ERK signaling, finally attributing to cellular alterations 20, 26, 27. As indicated in Figure 5A, diminished phosphorylation of AKT and ERK were detected in FLRT2-overexpressing HCT116 and SW480 cells. Upon PI3K activation, the 3-phosphoinositide-dependent protein kinase 1 (PDK1) directly phosphorylates AKT and play a key role in mediating many of the signaling functions 28. As expected, the level of phosphorylation of PDK1 was downregulated in CRC cells with FLRT2 overexpression. To explore the inhibitory effect of FLRT2 in cell growth and cell motility, the expression level of several important genes such as CDK, Cyclin A, P21, and MMP2, which is regulated by AKT activation, were detected. Data showed that FLRT2 overexpression resulted in a marked decrease in CDK2, Cyclin A and MMP2 while an increase in P21 (Figure 5B and 5C). These data together imply that FLRT2 functions as a tumor suppressor in CRC through AKT and ERK signaling pathway.

## Discussion

Epigenetic changes, especially the increased methylation variability, is a major driving force for CRC tumorigenesis and may be a valuable tool for cancer diagnostic and prediction of treatment response. In the present study, we identified aberrant methylated genes using MethylRAD-Seq to explore epigenetic biomarkers that contribute to CRC initiation and progression. Among the target genes, FLRT2 exhibited significantly higher methylation in CRC compared to normal tissues, which was further confirmed in CRC tissues and cell lines by MSP. And the promoter methylation of FLRT2 caused the decreased expression of FLRT2. We further clarified that FLRT2 might act as a tumor suppressor which suppressed cell growth and migration through mediating PI3K/AKT and ERK signaling pathways.

It has been reported that methylation marks are important molecular hallmarks of CRC, as they occur early in the progression of CRC 3. DNA methylation changes are not only abundant in CRC, but also have clinical importance. Changes in DNA methylation have shown promise as clinically relevant biomarkers for early diagnosis, prognosis and direction of therapy in CRC. In addition, DNA methylation alterations, which are reversible, make them attractive therapeutic targets. DNA methylation inhibitors have been tested preclinically or in early-phase clinical trials in CRC 2, 8, 29. In our present study, we found that numerous genes were aberrantly methylated, including FLRT2 (Figure 1). Intriguingly, FLRT2 hypermethylation was found not only in CRC tissues but also in normal mucosal epithelium, which implying that FLRT2 methylation may be an essential driver for CRC initiation. Further clinical relevance analysis revealed that methylation of FLRT2 is closely associated with tumor differentiation, lymphatic metastasis in CRC patients (Table 2), which strongly indicated that FLRT2 methylation is also involved in CRC progression. These findings greatly detail the potential clinical usefulness of FLRT2 methylation as biomarkers for diagnosis and management of CRC patients. It has been demonstrated that gene methylation patterns are regulated by DNA methyltransferases (DNMTs) and critical for gene regulation 30, 31. Additionally, dysregulation of histones and their modifying proteins may cause abnormal DNA methylation 5, 32, 33. Therefore, more detailed mechanisms about aberrant FLRT2 hypermethylation need to be further explored.

It is well known that promoter hypermethylation leads to aberrant expression of tumor suppressor genes, contributing to cell malignant transformation and the development of CRC 2, 3, 5, 34-37. Indeed, downregulated FLRT2 expression was observed in CRC tissues, accompanying with DNA methylation (Figure 2). *In vitro* experiments showed that FLRT2 functions as a tumor suppressor in CRC by suppressing cell proliferation and cell colony formation, and inhibiting migration and invasion. Furthermore, we showed that FLRT2 induced cell cycle arrest in G0/1 to affect cell growth *in vitro* (Figure 3 and 4). It has been demonstrated that FLRT2 can interact with FGF receptors, modulating PI3K/AKT and ERK signaling pathway, finally attributable to cellular alterations 20, 26, 27. Emerging evidence clearly indicates that PI3K/AKT and ERK signaling pathway participate in regulating cellular events, such as cell growth, migration and adhesion. Activation of these signaling molecules can contribute to cell proliferation and tumor progression by modulating downstream factors 38. Genetic inactivation of AKT results in reduction in clonal growth of CRC cells, reduced metastasis to liver, and reduced tumor burden 39. In fact, we found that phosphorylated AKT and ERK were downregulated with ectopic expression of FLRT2 in CRC cell lines. Akt has been shown to regulate a wide range of downstream targets which includes proteins central to the regulation of cell survival, cell cycle progression and cell motility. Here, we found that cell cycle markers, CDK2 and Cyclin A were downregulated with FLRT2 overexpression, while cyclin-dependent kinase inhibitor P21 increased. The level of MMP2 was also found decreased (Figure 5). These data indicate that FLRT2 exerts tumor suppressive roles through AKT and ERK signaling pathway. Future studies may provide additional information regarding the precise contribution of AKT and ERK signaling downregulation by FLRT2.

In conclusion, our findings highlight a tumor-suppressive role of FLRT2 in CRC progression, probably by regulating AKT and ERK signaling pathway. These observations suggest that FLRT2 methylation alterations can be exploited as predictive and diagnosis biomarkers for CRC metastasis and tumorigenesis, and also provide a therapeutic option for the disease.

## Figures and Tables

**Table 1:** The association of FLRT2 methylation status and expression in 22 patients with colorectal cancer

Group	N	FLRT2		*P*
		M	U	
FLRT2 low expression	16	13 (81.3)	3 (18.7)	0.011
FLRT2 high expression	6	1 (16.7)	5 (83.3)	

**Table 2:** Clinicopathological features and FLRT2 methylation status in 51 patients with colorectal cancer

Clinical parameters	N	FLRT2		χ^2^	*P*
		M	U		
**Age (years)**				1.152	0.283
>60	30	17	13		
≤60	21	15	6		
**Gender**				0.421	0.517
Male	35	23	12		
Female	16	9	7		
**Tumor differentiation**				4.191	0.041
Moderate and well	31	16	15		
Poor	20	16	4		
**Tumor size (cm)**				0.694	0.405
>5	23	13	10		
≤5	28	19	9		
**Tumor location**				0.61	0.435
Right	8	6	2		
Left	43	26	17		
**Lymphatic metastasis**				5.202	0.023
Positive	15	13	2		
Negative	36	19	17		

## Abbreviations

CRC: colorectal cancer
FLRT2: fibronectin leucine rich transmembrane protein 2
5-AZA: 5-Aza-2′-deoxycytidine
FGF: fibroblast growth factor
MSP: methylation-specific PCR
COAD: colon adenocarcinoma
READ: rectum adenocarcinoma
DNMTs: DNA methyltransferases
